# Maximizing Potential: Academic–Industry Collaborations in Drug Discovery

**DOI:** 10.1021/acsmedchemlett.5c00331

**Published:** 2025-07-03

**Authors:** Susan Winks, Jutta Reinhard-Rupp, Kelly Chibale

**Affiliations:** † Drug Discovery and Development Centre (H3D), University of Cape Town, Rondebosch, Cape Town 7701, South Africa; ‡ H3D Foundation, c/o DNDi, Chemin Camille-Vidart 15, 1202 Geneva, Switzerland; §Drug Discovery and Development Centre (H3D) and ∥South African Medical Research Council Drug Discovery and Development Research Unit, Department of Chemistry and Institute of Infectious Disease and Molecular Medicine, University of Cape Town, Rondebosch 7701, South Africa

**Keywords:** Academic−Industry Collaboration, Public−Private Partnership, Drug Discovery, Translational Research

## Abstract

Academic–industry collaborations are increasingly recognized as a catalyst for pharmaceutical innovation. While such partnerships are broadly valued across disciplines, their relevance to drug discovery is particularly pronounced due to the field’s interdisciplinary complexity, high cost, and inherent risk. Drawing on direct experience of academic–industry collaborations, we explore the benefits and challenges.

## Graphical abstract

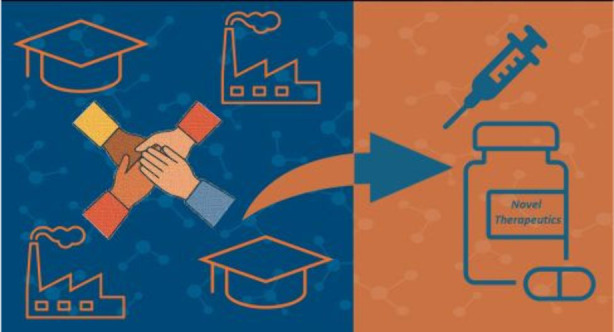

Academic–industry collaborations are broadly beneficial across scientific and non-scientific disciplines. On a base economic level, the benefit to industry is obvious, these collaborations achieve two critical and synergistic outcomes; first, it provides the workforce necessary to run the industry, and second, it furnishes innovative ideas to start new business ventures.^1^ It is also beneficial from an institutional perspective, where academic–industry collaborations are encouraged to support socioeconomic development,^2^ stimulating economic growth, and for financial gain for the universities.^3^ Furthermore, it provides academics practical problems to which they can apply their knowledge and allows the training of post graduate students to be of industrial relevance,^1^ increasing the employability of graduates. For individual academics, industry collaborations represent an attractive opportunity for securing research funds and resources, generating research results and potential personal financial reward (in the case of successful commercialization).^2^

However, the value and impact of academic–industry partnerships in drug discovery is even more pronounced, with academic–industry collaborative R&D viewed as a key driver of the pharmaceutical innovation process.^4^ The inherent reasons for this lie in the nature of drug discovery research, which is highly interdisciplinary, with different expertise and specializations required. Furthermore, the costs and risks associated with developing an innovative drug are unusually high compared to other innovations.

Drug discovery in academic environments faces three specific challenges: 1) lack of funding to bridge the translation gap between basic research and development; 2) lack of expertise within universities to evaluate translational potential of drug discovery assets; 3) a tendency to replicate the industrial model within academia on a small, inefficient scale owing to limited resources.^4^

Academic–industry collaboration offers the benefit of risk sharing and reduces the associated cost for each of the collaborators.^5^ The partnerships have the added benefit of reducing the time involved by leveraging existing infrastructure and expertise at different sites and increasing the flexibility to respond and adapt to the evolving scientific questions. The Holistic Drug Discovery and Development Centre, H3D, at the University of Cape Town in South Africa, has directly benefited from multiple industry partnerships, which have been a key element of its success to date and serves as a model for drug discovery and development research in Africa.^6^

While it is easy to appreciate the benefit of academic–industry partnerships, building and sustaining effective partnerships takes time and commitment from both parties. The main pillars of partnership are developing trust and rapport, being clear on expectations from each partner and strong communication. It takes time to develop the necessary interpersonal relationships; in the initial stage of relationship building, individual trust can be established based on professional reputation and shared background.^3^ Organizational trust is typically linked to organizational reputation, and the engagements during the formal contracting processes play a pivotal role in developing a baseline of trust.^3^ During these early stages, regular social interactions or the use of intermediaries can help pave the way.^3^

The divergent missions of universities and industrial organizations result in an array of cultural differences that need to be navigated. Universities are typically focused on training students and pursuing research that creates and disseminates new knowledge, whereas industrial organizations have focused research programs aiming to commercialize or exploit the knowledge as soon as possible.^3^ For industry, the degree of regulation by law and authorities is very high, which is not the case for academia. This difference in mission and regulation permeates through organizational design, policies, governance structures and organizational culture, and both parties need to be committed to a collaboration framework to support the needs of both institutions. Strong project management, clear communication and good governance structures are essential components for building a robust collaborative team. The team members from both parties should strive to contribute equally, support team decision making and navigate potential power dynamics to ensure that emerging researchers or students working on the project are empowered to speak up and share scientific insights. However, while conflicting organizational cultures is a significant hurdle to overcome, the most frequent challenges for academic industry partners relate to legal and administrative process complexity, resource constraints, coordination and scientific challenges.^7^

Establishing formal legal agreements between academic institutions and industry partners can present an array of challenges, particularly if either one of the parties has not engaged in these types of collaborations in the past. There could be a strong alignment of intention for a joint scientific collaboration, but the nature of the organizational policies and governance differs significantly, which can result in drawn out and bureaucratic negotiations to meet the requirements of both parties. Nevertheless, care should be taken as the legal framework is crucial for preventing potential misappropriation of technological and strategic knowledge and for laying the foundation for the partnership.^3^ If possible, the parties should strive to establish reciprocity in the agreement, ideally with an element of cofunding by the academic institution. Furthermore, the legal agreement should ensure that the process of defining the IP rights is collaborative and inclusive.^3^ Interestingly, Gersdorf et al.^7^ found that projects involving more complex legal contracts (e.g., collaboration agreements rather than data transfer agreements) reported greater success, and while complex legal contracts take time to put in place, once the project is running, they do not encumber collaboration.

In our experience, a sound legal framework, joint project governance, and strong project management are essential components for a sustainable partnership.

One of the considerations for academic–industry partnerships focused on drug discovery is the volatile nature of the industry. The innovative pharmaceutical industry tends to be exposed to big changes; mergers, acquisitions, changes in leadership, changes in strategy and restructuring with the resulting effect of staff turnover and instability. This adds pressure to the individuals involved in the academic and industry partnership to develop a strong relationship and formalize the partnership as soon as feasible; otherwise, the opportunity to collaborate can be lost.

Once the legal partnership is in place, the relationship is still at the mercy of the larger pharma industry movements with shifting strategies and priorities. In our experience the partnerships can sometimes end abruptly despite steady progress in the joint project. To mitigate this, the parties should ensure that there is a clear exit plan built into the formal agreements to help preserve any assets developed during the partnership in the event of early termination. This could involve licensing or royalty agreements, IP assignment, first right of refusal or other suitable mechanisms. Whichever route is agreed on, it should include a clear pathway for development of the assets beyond the partnership and a plan to secure longer term involvement of the academic partners in case of PhD students who need to complete their thesis.

In drug discovery, academic institutions represent fertile environments for innovation, new drug discovery targets, new tools, new strategies, new approaches emerging on the back of academic research projects and a prime environment to nurture translational drug discovery projects. The academic environment encourages cross pollination of ideas between researchers from different disciplines and different institutions, and the academic partner will usually have a particularly well-developed research niche ripe for exploitation. On the other hand, the industry partner will typically have access to greater resources, funding, and be able to tap into drug development expertise, including toxicology, regulatory, formulation, pharmacology and clinical expertise. The opportunity for the parties to join forces, pool resources, expertise and networks is apparent, which in turn serves to accelerate innovation.

The broader and more diverse the collaboration, the greater the opportunity for consensus building through many different viewpoints coming together, ultimately supporting the right questions being raised and addressed within the team.^5^ This is particularly relevant in drug discovery research, where the scientists are each expert in their own niche and are more likely to make good decisions and avoid costly errors if they work together.^5^

In addition to the knowledge exchange that is part and parcel of any collaboration, one of the key benefits to academic institutions is the automatic training and capacity building that comes on the back of industry academia partnerships, based on inherent exposure of the team members to interdisciplinary problem solving. The industry mindset supports rapid decision making to kill a project or take it forward as fast as possible. This is because delayed decision making drives up project costs. This is very different from the typical academic mindset which focuses on understanding the problem at hand as fully as possible, exploring different angles and pursuing optimum results. The industryexposure that science graduates can benefit from includes rapid decision making, cost benefit analysis, risk assessments, portfolio prioritization, competitive analysis, clarity around the target product profile, and an understanding of the route to market. The science graduates also gain perspective on the potential job market, which prepares them for employment and serves to attract more students into relevant fields.^7^

H3D has been privileged to be involved in numerous academic industry partnerships, including partnerships with big pharma and partnerships with smaller specialized companies. In all cases the impact of the partnership has led to pooling of resources, faster decision making, and synergies in terms of capabilities and expertise. While Cantner et al.^2^ posit three main benefits to industry–academia collaboration as 1) scientific outcomes, 2) commercial outcomes, and 3) follow-up cooperation, in our experience the benefit to H3D included raising the profile of the center, upskilling more scientists through on-the-job training, local job creation, technology transfer or incorporation of new techniques and technology at H3D, risk sharing, and increased foreign direct investment for South Africa.

We support Cantner et al.^2^ in their conclusion that most scientists and industry managers engaging in industry academia collaborations confirmed the benefit of the partnership, reported positive experience, and expressed a desire to maintain or extend their level of engagement with the other party.

Our personal experience at the H3D center is echoed by a recommendation from Rossoni et al.^8^ that collaborative barriers may be overcome by starting with smaller projects and gradually increasing their complexity. This helps to build trust and social capital between the partners and to develop a strong working partnership before tackling complex collaborative projects together. Another method for increasing trust is to adopt multiple channels for interactions,^3^ to implement flexible and transparent IP policies, to share scientific governance, and to champion behavior that enables the formation of trust.^3^

Once the partnership has been formalized, strong project management skills to support effective communication and objective setting and alignment are critical, and if the project manager understands the challenges of both parties, this will enhance the project success.^3^ The project manager should support the team to flexibly adapt to changes in strategy or project direction, while balancing the needs of both parties. This includes particular consideration if students are included in the project team to ensure their academic needs, including publication strategies, are fulfilled.^3^

Overall, high levels of flexibility and adaptability, high personal commitment, and implementing standard project management practices like regular project meetings, open discussions, and conflict resolution are essential for a successful partnership.^7^ If an industry academia collaboration is truly successful, the result will be lower research and development costs and higher levels of innovative output.^3^

Our personal recipe for success at H3D is: mutual interest, mutual responsibility, mutual accountability, mutual trust, mutual respect and shared goal/vision. For the academic–industry partnership to succeed, there must be clear benefit to both partners and strong alignment on the collaboration objectives. If these ingredients are present then, with sound governance, clear communication plans, strong project management, and commitment from both sides, there can be unquantifiable benefits, from fueling innovation, training scientists, and ultimately improving patient outcomes.

## Safety

No unexpected or unusually high safety hazards were encountered.

## Abbreviations

R&D: Research and Development
IP: Intellectual Property
H3D: Holistic Drug Discovery and Development Centre
